# It’s time to develop a clinical guideline for ChatGPT usage

**DOI:** 10.1097/JS9.0000000000001576

**Published:** 2024-05-08

**Authors:** Haiyang Wu, Zaijie Sun, Cheng Li

**Affiliations:** a Department of Orthopaedics, The First Affiliated Hospital of Zhengzhou University, Zhengzhou; b Department of Clinical College of Neurology, Neurosurgery and Neurorehabilitation, Tianjin Medical University, Tianjin; c Department of Orthopaedics, Xiangyang Central Hospital, Affiliated Hospital of Hubei University of Arts and Science, Xiangyang; d Department of Orthopaedic Surgery, Beijing Jishuitan Hospital, Capital Medical University, Beijing, People’s Republic of China; e Center for Musculoskeletal Surgery (CMSC), Charité-Universitätsmedizin Berlin, Corporate Member of Freie Universität Berlin, Humboldt University of Berlin, Berlin Institute of Health, Berlin, Germany

*Dear Editor,*

The advent of ChatGPT has been hailed by many experts as a revolutionary technology, marking the dawn of a new era in artificial intelligence (AI). Many journals including *International Journal of Surgery* have noticed the huge potential of ChatGPT and published multiple papers^1,2^. From crafting scientific papers, coding, to passing the United States Medical Licensing Examination (USMLE), ChatGPT continually demonstrates remarkable versatility across various domains. Recently, there has been an increasing body of research that attempts to explore the potential applications of ChatGPT in various medical disciplines such as urology, oncology, neuroscience, and obstetrics and gynecology^3,4^. For example, one study published in *Science* also demonstrated that ChatGPT could enhance productivity, particularly benefiting individuals with poor writing and communication skills^5^. By providing powerful creative generation skills and the ability to articulate ideas, ChatGPT enhances competitiveness in the labor market.

However, with the deepening of research, controversies have emerged regarding the application of ChatGPT, particularly with its ethical implications being paramount. On the one hand, there are concerns that ChatGPT might be employed to mislead, deceive, or manipulate individuals, particularly in the realms of social media and online platforms. On the other hand, apprehensions regarding how ChatGPT handles personal data and privacy are increasingly pronounced. These controversies have spurred calls for regulatory oversight and transparency in the application of ChatGPT, aiming to ensure its use in society is both responsible and ethical.

In addition, there is a significant problem that requires attention: some published studies may have methodological flaws and limitations. These issues may affect the objectivity and reliability of the research results, leading to varying degrees of bias. Especially in different generative AI models, the situation that may produce different responses to the same query is more prominent. Changing the way a question is asked, or even just repeating a question slightly, could cause ChatGPT to give completely different answers. Another important gap lies in the lack of an established framework or guideline to systematically evaluate the ability of generative AI to solve problems and apply solutions in clinical medicine. Therefore, it is crucial to develop a comprehensive set of assessment guidelines. Such a framework should cover key dimensions, including question collection, questioning methods, and diverse assessment methods. Through such a framework, not only could the evaluation process be standardized, but also research on the application of generative AI in the medical field could be significantly promoted.

To our understanding, when developing a comprehensive clinical guideline for ChatGPT usage, several key factors must be taken into consideration: First, the question collection phase should be the basis for the evaluation. This includes determining the importance and relevance of the problem and gathering data and literature related to it. Second, the method of asking questions is crucial. Different ways of asking questions may result in different types and quality of responses. Therefore, when designing questioning methods, the clarity, logic, and completeness of the questions need to be taken into account to ensure that the answers obtained are comparable and credible. Finally, diversification of assessment methods is also crucial. Accuracy is an important metric, but completeness and readability are equally important. Generative AI models not only need to give accurate answers, they also need to be able to provide comprehensive information and present it to users in an easy-to-understand way. Therefore, the assessment method should cover these aspects to ensure the comprehensiveness and objectivity of the assessment.

In summary, developing a comprehensive assessment guideline is critical to advancing the application of generative AI in clinical medicine. Such a framework could not only help correct possible biases and flaws in existing research but also guide the design and implementation of future research to better meet the needs of the medical field and promote the development and advancement of health care.

## Ethical approval

This study does not include any individual-level data and thus does not require any ethical approval.

## Sources of funding

This study is supported by China Postdoctoral Science Foundation (2022M720385) and Beijing JST Research Funding (YGQ-202313).

## Author contribution

H.W.: conceptualization, methodology, data curation, formal analysis, resources, investigation, and writing – original draft; Z.S.: conceptualization, methodology, data duration, formal analysis, resources, and investigation; C.L.: methodology, data duration, formal analysis, resources, investigation, and writing – review and editing.

## Conflicts of interest disclosure

The authors declare no conflicts of interest.

## Research registration unique identifying number (UIN)

1. Name of the registry: not applicable.
2. Unique identifying number or registration ID: not applicable.
3. Hyperlink to your specific registration (must be publicly accessible and will be checked): not applicable.

## Guarantor

Haiyang Wu and Cheng Li.
